# Effective ecosystem monitoring requires a multi‐scaled approach

**DOI:** 10.1111/brv.12636

**Published:** 2020-07-09

**Authors:** Ben D. Sparrow, Will Edwards, Samantha E.M. Munroe, Glenda M. Wardle, Greg R. Guerin, Jean‐Francois Bastin, Beryl Morris, Rebekah Christensen, Stuart Phinn, Andrew J. Lowe

**Affiliations:** ^1^ Terrestrial Ecosystem Research Network, The School of Biological Sciences The University of Adelaide Adelaide South Australia 5005 Australia; ^2^ Terrestrial Ecosystem Research Network, College of Science and Engineering James Cook University PO Box 6811 Cairns Queensland 4870 Australia; ^3^ Terrestrial Ecosystem Research Network, Desert Ecology Research Group, School of Life and Environmental Sciences University of Sydney Sydney New South Wales 2006 Australia; ^4^ Computational and Applied Vegetation Ecology Lab, Department of Applied Ecology and Environmental Biology, Faculty of Bioscience Engineering Ghent University Ghent 9000 Belgium; ^5^ Terrestrial Ecosystem Research Network The University of Queensland St Lucia Queensland 4072 Australia; ^6^ Institute for Future Environments Queensland University of Technology Gardens Point Brisbane Queensland 4000 Australia; ^7^ School of Earth and Environmental Sciences The University of Queensland St Lucia Queensland 4072 Australia; ^8^ School of Biological Sciences The University of Adelaide Adelaide South Australia 5005 Australia

**Keywords:** ecosystem monitoring, targeted monitoring, surveillance monitoring, landscape monitoring, ecological questions, environmental change, biodiversity monitoring, research infrastructure, collaboration, environmental monitoring

## Abstract

Ecosystem monitoring is fundamental to our understanding of how ecosystem change is impacting our natural resources and is vital for developing evidence‐based policy and management. However, the different types of ecosystem monitoring, along with their recommended applications, are often poorly understood and contentious. Varying definitions and strict adherence to a specific monitoring type can inhibit effective ecosystem monitoring, leading to poor program development, implementation and outcomes. In an effort to develop a more consistent and clear understanding of ecosystem monitoring programs, we here review the main types of monitoring and recommend the widespread adoption of three classifications of monitoring, namely, targeted, surveillance and landscape monitoring. Landscape monitoring is conducted over large areas, provides spatial data, and enables questions relating to where and when ecosystem change is occurring to be addressed. Surveillance monitoring uses standardised field methods to inform on what is changing in our environments and the direction and magnitude of that change, whilst targeted monitoring is designed around testable hypotheses over defined areas and is the best approach for determining the causes of ecosystem change. The classification system is flexible and can incorporate different interests, objectives, targets and characteristics as well as different spatial scales and temporal frequencies, while also providing valuable structure and consistency across distinct ecosystem monitoring programs. To support our argument, we examine the ability of each monitoring type to inform on six key types of questions that are routinely posed for ecosystem monitoring programs, such as where and when change is occurring, what is the magnitude of change, and how can the change be managed? As we demonstrate, each type of ecosystem monitoring has its own strengths and weaknesses, which should be carefully considered relative to the desired results. Using this scheme, scientists and land managers can design programs best suited to their needs. Finally, we assert that for our most serious environmental challenges, it is essential that we include information from each of these monitoring scales to inform on all facets of ecosystem change, and this is best achieved through close collaboration between the scales. With a renewed understanding of the importance of each monitoring type, along with greater commitment to monitor cooperatively, we will be well placed to address some of our greatest environmental challenges.

## I. INTRODUCTION

Our natural and managed ecosystems are changing more dramatically than at any other time in human history. Habitat destruction, soil erosion, pollution, climate change and species extinction (together with and loss of genetic resources) are just some of the most serious environmental problems facing our planet (Diamond, 2005; Warner *et al*., 2010). The impacts of these problems are not only environmental, they also have huge economic consequences due to reductions in the provision of ecosystem services (Costanza *et al*., 1997; de Groot *et al*., 2012; Kubiszewski *et al*., 2017) – including clean water, productive lands for food and essential pollination services – valued at trillions of dollars per year (Costanza *et al*., 2014).

To address both environmental and economic decline, natural resource managers and policymakers need to make informed decisions when developing management actions. We need to understand what, where, when, and how much change is occurring, in order to develop an understanding of the causes of change, and thus generate actions that will mitigate its potentially negative effects. The fundamental information required for best‐practice decision making can only be achieved through dedicated monitoring programs that occur in sufficient detail and at appropriate spatial and temporal scales to inform planning, design, and budgeting phases (Smyth & James, 2004; Pettorelli *et al*., 2014*a*). Clearly, there is a strong need for ecosystem science to deliver tools, data and knowledge to respond in an informed way to current and future environmental challenges (Andersen *et al*., 2014) and to support the development of evidence‐based policy and management (Eyre *et al*., 2011).

Ecosystem monitoring programs have the ability to provide essential information on ecosystem drivers (Vos, Meelis, & Ter Keurs, 2000; Peters *et al*., 2014) and to clarify the dynamics of environmental systems (Burton *et al*., 2014; Lindenmayer *et al*., 2014). Other major applications and/or outcomes of ecosystem monitoring programs include measuring biodiversity change (Pereira *et al*., 2013), providing early warning of significant environmental change (Landsberg & Crowley, 2004), identifying changes in condition and biodiversity loss (Turner, 2014), and identifying tipping points and thresholds (Huete, 2016). Monitoring allows us to assess the impact of management actions (Failing & Gregory, 2003; Lindenmayer, Piggott, & Wintle, 2013), the return on investment of management actions and policy (Lindenmayer & Likens, 2010; Lindenmayer *et al*., 2012), when to implement conservation actions (Lindenmayer *et al*., 2013) and the impacts of climate change (Abbott & Le Maitre, 2009). In short, continuous, long‐term, monitoring is essential to report reliably on the status of ecosystem assets and environmental condition trends (Woinarski, 2012).

Despite the widely recognised importance of effective ecosystem monitoring, there is surprisingly little agreement on what types of monitoring can enable us to address these key questions. Some authors delineate different monitoring types based on the underlying motivation of the program. For example, monitoring programs may be curiosity driven (devoid of questions), mandated (data collected as a legislative requirement) or question driven (guided by a conceptual model and rigorous study design) (Lindenmayer & Likens, 2010). Alternatively, other classifications are distinguished based primarily on scale and purpose, such as targeted (regional scale, detects process‐based change), surveillance (regional to national scale, detects location, magnitude, and direction of change) and landscape (national scale, detects spatially continuous change) monitoring (Eyre *et al*., 2011). Monitoring can also be stratified based on its aims or intended management outcomes, such as implementation monitoring – which determines if management actions were applied as prescribed; effectiveness monitoring – which evaluates if management action was effective in meeting a stated objective; and effects monitoring – which can reveal the unintended ecological consequences of management actions (Hutto & Belote, 2013). Amidst these numerous classifications, an additional complexity is that many authors are staunch advocates for a particular type of monitoring (Legg & Nagy, 2006; Nichols & Williams, 2006; McDonald‐Madden *et al*., 2010; Lindenmayer *et al*., 2012, 2015).

In this review, we define and describe different monitoring types in order to support what we argue is an inclusive, consistent, and useful classification scheme for monitoring activity, namely targeted, surveillance, and landscape monitoring as first described in Eyre *et al*. (2011). To illustrate the value of this system and each form of monitoring, we review the types of environmental, ecological and ecosystem questions commonly asked of monitoring systems. We then outline the strengths and weaknesses of each monitoring type, before exploring the benefits of combining different monitoring programs into a coherent monitoring system, and the value that each component brings to this holistic program. We demonstrate that different forms of monitoring are required (Ferrier, 2012) to address and inform on a variety of environmental decision‐making processes and that environmental information should be collected on a variety of processes that vary in space and time to make effective and productive management or conservation decisions (Andersen *et al*., 2014).

## II. MONITORING QUESTIONS AND CLASSIFICATIONS

Each year we expect ecosystem monitoring programs to answer a great number of questions and/or test and evaluate a range of hypotheses. Whilst specific questions embedded within monitoring programs may be taxon, environment and location specific, we argue that there are six fundamental categories of questions that scientists and managers routinely consider, and that ecosystem monitoring data must be capable of answering. These are: (*i*) what elements within the environment are changing? (*ii*) What is the direction and magnitude of that change? (*iii*) Where is environmental change occurring in the landscape? (*iv*) When is environmental change occurring and is the rate of change increasing or decreasing? (*v*) What is the cause of the environmental change we are observing? (*vi*) What action can be taken to ameliorate deleterious change and/or encourage positive change?

Inconsistent definitions and rigid adherence to particular approaches can be serious barriers to effective ecosystem monitoring, leading to poor program design, execution, and results. In reality, each type of ecosystem monitoring has its own strengths, weaknesses, and applications (Hutto & Belote, 2013), all of which must be carefully considered relative to the desired outcomes. By working in concert, different types of monitoring strategies can provide the complementary information required to assess and examine ecosystem change at various scales, allowing managers to address a diverse range of objectives and questions.

To help understand how different types of monitoring can provide essential information to deal with the significant challenges we have identified, we advocate for the classification of ecosystem monitoring into the framework of targeted monitoring, surveillance monitoring and landscape monitoring first described in Eyre *et al*. (2011). *Targeted monitoring* is used to describe local to regional monitoring, with several re‐visits per year, designed with the aim of understanding ecosystem processes occurring in particular environments. *Surveillance monitoring* is designed to detect when change is occurring, what that change is and the magnitude of that change, using standardised methods to collect a broad suite of variables at regional to national scales. *Landscape monitoring* is conducted over large areas, provides spatially continuous data and is primarily concerned with where and when change is occurring and provides information that cannot be feasibly collected using other methods.

Here we argue that the classification framework proposed by Eyre *et al*. (2011) is the most appropriate and broadly applicable system to date that can address all of the questions identified above. We have three primary reasons for supporting this framework; first, this classification system is highly inclusive and can incorporate different motivations and goals, including curiosity‐driven programs or those designed to observe management effects. As a result, program intent and purpose can easily be considered within the system. Second, the different types of monitoring within this system are primarily distinguished by different spatial scales, temporal frequencies, and the ecological information content contained within them, allowing scientists and managers to determine the scale at which each type of monitoring is most effective. Finally, using this structure, users can assess the ability of different types of ecosystem monitoring to inform on different ecological questions. This is important because, as will be discussed later, each type of monitoring contributes information capable of answering different potential questions. In sum, this classification system is both comprehensive and flexible.

## III. AN EXPLANATION OF MONITORING TYPES

### (1) Targeted monitoring

Targeted monitoring programs focus on discreet areas (typically the site or regional level) and are generally designed to address a specific hypothesis (Lindenmayer *et al*., 2012) (see Table 1). Hypotheses underlying targeted monitoring are based on an understanding of the variation in and drivers of the ecosystems being investigated, often with the assistance of a conceptual model (Lindenmayer & Likens, 2010). Targeted monitoring is also referred to as question‐based monitoring (Lindenmayer & Likens, 2010), long‐term monitoring (Lindenmayer *et al*., 2012), or simply ‘monitoring’ (Vos *et al*., 2000; Legg & Nagy, 2006; McDonald‐Madden *et al*., 2010), although other types of monitoring can be both question‐driven and long term. Targeted monitoring is the most widely accepted and utilised form of ecosystem monitoring, so much so that the term ‘monitoring’ is often used synonymously with targeted monitoring (Yoccoz, Nichols, & Boulinier, 2001). Targeted monitoring generally focusses on population‐level responses (Eyre *et al*., 2011) of individual species or a relatively small subset of species, with the intent of investigating the interactions between them. This makes targeted monitoring a powerful way to detect and test expected or predicted change in an environment, and to inform on the cause of that change (Wintle, Runge, & Bekessy, 2010). An example of one such program is the work of Buitenwerf *et al*. (2012) in Kruger National Park (50 year timeseries) and the Eastern Cape Province (30 year timeseries) of South Africa. Buitenwerf *et al*. (2012) investigated if increased levels of atmospheric CO_2_ concentration, the effects of fires, or grazing pressure were correlated with increased tree densities in savanna systems and concluded that the most likely cause was increasing atmospheric CO_2_, an outcome explicitly revealed as a function of their survey design that utilised timeseries data (before and after intervention) and paired sites to assess the effect of fires and grazing by including control plots.

**Table 1:** Key traits of different monitoring types

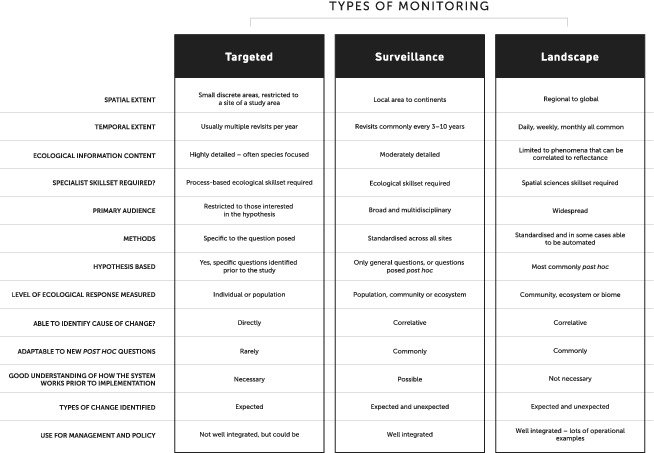

Targeted programs can also focus on understanding the cause of local change and can help to determine the preferred action from several viable alternatives. By examining processes within an ecosystem, targeted monitoring programs are able to inform on the likely causes of change in similar ecosystems in other areas. Bestelmeyer *et al*. (2018) used a series of research outcomes from the Jornada Basin Long Term Ecological Research (LTER) site to understand the cause of change in the North American desert grassland region and suggest appropriate management interventions. Research at the Jornada Basin LTER site showed that increased seed dispersal and decreased fire frequency were associated with livestock grazing pressure (particularly after extended drought conditions). This then enabled shrubs to outperform perennial grasses (Van Auken, 2009), largely driven by aridity and increases in atmospheric carbon. Management interventions recommended as a result of this work included dynamically managing livestock relative to rainfall to encourage grass recovery, and the use of herbicide and prescribed fire to reduce shrub cover (Coffman *et al*., 2014; Bestelmeyer *et al*., 2018).

Because this type of monitoring is most often focused at the site or regional level, it is difficult to generalise results to different environments, given the specific nature of the hypotheses addressed. Targeted monitoring programs are usually designed as ‘one‐off’ studies that rarely produce information at the scale governments require for regional or continental decision making (Lindenmayer & Likens, 2010). As a result these studies are most commonly conducted by an individual research laboratory, a single branch of a government department or management agency, or localised land managers, although networks of studies fit well with research infrastructure programs (Peters *et al*., 2014; Cleverly *et al*., 2019).

Targeted monitoring data‐collection methods and protocols are commonly site specific, and therefore may not be applicable to other targeted monitoring programs at alternative locations. Targeted monitoring may also include a high re‐visit frequency of multiple re‐visits per year (Dickman *et al*., 2014). This is because targeted monitoring is designed with the aim of understanding processes occurring in particular environments (Dickman & Wardle, 2012). These studies require regular and ongoing sampling efforts to quantify change, necessitating numerous surveys per year over long time periods, especially in environments with unpredictable climate variability (Dickman *et al*., 2014). The duration and effort invested in a targeting monitoring program hinges on the question itself, the location(s), the number of variables being considered, and the expected rate of change (Lengyel *et al*., 2018; Lang *et al*., 2019) in target species or factors. Targeted monitoring, in particular hypothesis‐driven programs, can benefit enormously from careful consideration of the sample size or time frame necessary to observe ecologically meaningful and significant change and test key objectives (Toft & Shea, 1983; Green, 1989; Fairweather, 1991).

Targeted monitoring studies do, however, need to ensure the methods of data collection remain constant through time to avoid potential confounding effects (Lindenmayer *et al*., 2015). Targeted monitoring programs are less flexible than other forms of monitoring, and the data derived from them cannot easily be re‐used or re‐purposed to answer other questions not considered in the original study (Wintle *et al*., 2010) and it is particularly difficult to combine independent targeted studies to examine trends at continental or global scales (Bunce *et al*., 2007).

### (2) Surveillance monitoring

Surveillance monitoring is primarily concerned with identifying what is changing in the environment and detecting the magnitude and direction of that environmental and ecosystem change through time (Watson & Novelly, 2004; Gillan *et al*., 2014) (see Table 1). Surveillance monitoring programs generally collect field data on a broad suite of biotic and abiotic variables that can inform on trends in species composition and relative abundances, and are known to be responsive to environmental change (Smith, 2002; Boutin *et al*., 2009; Eyre *et al*., 2011; Kao *et al*., 2012; Pereira *et al*., 2013; La Salle, Williams, & Moritz, 2016; Guerin *et al*., 2017). Surveillance monitoring is also referred to as omnibus monitoring (Nichols & Williams, 2006), passive monitoring (Hutto & Belote, 2013), mandated monitoring (Lindenmayer & Likens, 2010), and biodiversity monitoring (Smyth & James, 2004; Watson & Novelly, 2004; Boutin *et al*., 2009). Surveillance monitoring commonly spans entire ecosystems, communities, or larger areas, often occurring across jurisdictional boundaries (Watson & Novelly, 2004; Hutto & Belote, 2013). Because of its broad scope and intent, surveillance monitoring is perhaps the least well‐understood type of monitoring. Nevertheless, given the wide range of variables and the extensive areas over which data are collected, surveillance monitoring is applicable to a broad stakeholder base.

The broad suite of possible variables that could be collected by surveillance monitoring creates the potential for an unlimited set of possible data attributes to be collected. Measuring all imaginable attributes is logistically impossible, however, and a process to identify important and generally useful variables must be undertaken. Pereira *et al*. (2013) suggested that data should be collected on a range of variables that can be categorised by genetic composition, species populations, species traits, community composition, and ecosystem structure and function. Based on the framework suggested by Pereira *et al*. (2013), the Group on Earth Observations Biodiversity Network (GEO BON) proposed a set of essential biodiversity variables (EBVs) that inform on the major dimensions of change. For example, community composition could be informed by consistent multi‐taxa surveys and metagenomics at select locations (Pereira *et al*., 2013). There is widespread support for the EBV approach (Scholes & Biggs, 2005; Scholes *et al*., 2008; Proenca *et al*., 2017; Haase *et al*., 2018; Kissling *et al*., 2018).

Numerous authors have argued that by tracking a wide suite of indicator variables at local to regional scales, measurements can be used to answer a broad range of questions at national, continental, and global scales. Programs designed to collect a wide suite of soil, vegetation and fauna data and samples are well positioned to feed into this EBV framework. Programs such as the Ecological Surveillance program (previously Ausplots) of Australia's Terrestrial Ecosystem Research Network (TERN) (Sparrow *et al*., 2020) and the MARAS system (Oliva *et al*., 2019) in Patagonia collect information on a wide range of variables. The MARAS program is particularly notable as a cross‐jurisdictional large‐scale environmental monitoring program because it requires international cooperation (Argentina and Chile) for the program's successful operation. Similarly, Herrick *et al*. (2010) describe a system that collects a wide range of soil and vegetation attributes throughout rangeland areas in the USA.

A key feature of surveillance monitoring programs is that methods are collected in a standardised manner, resulting in directly comparable data sets spanning large spatial scales (Belovsky *et al*., 2004; Bunce *et al*., 2007; Borer *et al*., 2014; Burton *et al*., 2014; Guerin *et al*., 2017). Standardisation enables the comparison and broad‐scale assessment of the dynamics of different ecosystems (Wood *et al*., 2015) in a way that facilitates continental‐scale questions to be addressed. Surveillance re‐visit periods in the order of years to decades are most common (Watson & Novelly, 2004). Hence, surveillance monitoring is particularly suited to detecting long‐term change over large areas. In common with targeted schemes, ensuring identical protocols are employed over the duration of a long‐term surveillance study (Lindenmayer *et al*., 2015) maximises the ability to extract signals of environmental change.

The real strength of surveillance monitoring programs is their ability to detect environmental change at a scale that enables regional and continental assessment (Stevens, 1994; Watson & Novelly, 2004; Wood *et al*., 2015; Guerin *et al*., 2017; Sparrow *et al*., 2020). The current lack of widespread systematic surveillance monitoring impedes broad‐scale analysis of change in the environment (Bastin *et al*., 2009). Unlike targeted monitoring schemes, surveillance monitoring programs are not explicitly designed to determine the biological processes that cause change, but this can emerge as an outcome of a statistical analysis of subsets of data once change has been detected. Furthermore, the resources required to cover the spatial extent of surveillance monitoring usually precludes the high‐frequency sampling required under targeted schemes (Kao *et al*., 2012). Surveillance monitoring collects information to enable change detection on a wide variety of variables more so than identifying the cause, and as such, it is not essential to have a complete understanding of the ecosystem to conduct effective monitoring (Wallace, Caccetta, & Kiiveri, 2004). These broadly focused programs can be effective even under circumstances where the environmental drivers for the system are not fully known (Hutto & Belote, 2013). Over time they build the knowledge base to inform strategic use of targeted monitoring to underpin the mechanistic understanding of environmental change. Further, given the scale at which these programs operate, they are ideal to include in fundamental national research infrastructure programs, with many programs internationally already doing this (Herrick *et al*., 2010; Toevs *et al*., 2011; Peters *et al*., 2014; Cleverly *et al*., 2019; Oliva *et al*., 2019; Sparrow *et al*., 2020).

When using surveillance monitoring, questions are often formulated *post hoc* and analysed using the pre‐existing data (Bayne, Stralberg, & Nixon, 2015; Guerin *et al*., 2017). Because surveillance monitoring programs produce data on a wide range of potential variables, often coupled with well‐curated environmental samples (Sparrow *et al*., 2020), data can be used in analyses that were not anticipated when the program was conceived (Wallace *et al*., 2004; Andersen *et al*., 2014). Instead, specific questions are detailed at the time of data analysis (Bastin *et al*., 2017; Guerin *et al*., 2017). Some authors (Nichols & Williams, 2006; Lindenmayer & Likens, 2010) view the lack of focus on specific questions as a weakness of surveillance monitoring because this method is not designed around a specific set of testable hypotheses, but see Lindenmayer *et al*. (2015) who ask what data should be collected now to answer the questions of the future. By contrast, Gibbons (2012) suggests that while strong data collection motivations must exist, they do not necessarily need to be in the form of *a priori* hypotheses. Indeed, reuse of data is a key principle underpinning findable, accessible, interoperable and reusable (FAIR) data (Wilkinson *et al*., 2016; Wilkinson *et al*., 2019) and many surveillance monitoring programs invest in making the data openly available for this reason. Furthermore, Hutto & Belote (2013) consider the suggestion that surveillance monitoring is not able to answer questions as spurious. Data derived from surveillance monitoring under a robust sampling design can address hypotheses formulated *post hoc* and are often able to infer processes underlying change through correlative approaches.

We agree that dismissing surveillance monitoring on the basis that it is not strictly hypothesis‐driven neglects the opportunities that this type of monitoring can provide, potentially leading to missed opportunities for discovery and insight that would otherwise not be made under a targeted monitoring framework (Wintle *et al*., 2010). An example is Lemetre *et al*. (2017) who investigated environmental factors associated with large‐scale variation in the community composition of therapeutically relevant bacterial bioactive metabolites found in soils. The study utilised soil samples collected across Australia as part of a national surveillance monitoring program and determined that the greatest compositional change was explained by latitudinal variation. Samples analysed in this study were not taken with this intent, but rather to supply the perceived increase in researcher demand for soil samples (Sparrow *et al*., 2020) for analysis of their microbiome as well as the potential for future environmental DNA sampling methods (Jarman, Berry, & Bunce, 2018). Nevertheless, without surveillance monitoring such as this, the pattern described by Lemetre *et al*. (2017) would never be revealed. This example demonstrates that surveillance data has a fundamental role in ecological research (Wintle *et al*., 2010). Most surveillance monitoring programs incorporate an adaptive management program review at regular intervals to improve procedures and incorporate new technologies where practical whilst maintaining compatibility with earlier data.

Using surveillance monitoring, key drivers of ecosystem change can be inferred through the correlation of observed change with a wide range of environmental variables or known management actions (Hutto & Belote, 2013). These correlations are strengthened by having a large number of sites, which is a general feature of surveillance monitoring networks (Bennett *et al*., 2014). Furthermore, when surveillance programs are designed to include paired benchmark (sites with minimal management interventions) and impacted (where change is influenced by management actions) sites, the site network can partition background site‐level specificity (stochastic variability associated with individual site location) from true directional environmental change (Landsberg & Crowley, 2004). Surveillance monitoring also has a role in informing the design of more mechanistically focused targeted monitoring programs (Hutto & Belote, 2013); it can identify trends that can then be investigated with a hypothesis‐driven framework to determine causation or allow causation to be extrapolated from similar sites.

### (3) Landscape monitoring

Landscape monitoring is primarily concerned with where and when change is occurring at very large spatial scales (including national, continental and global), and is principally focussed on analysis of vegetation communities or biomes (Pettorelli *et al*., 2014*a*) (Table 1). Thus, it is the broadest spatial scale of all monitoring types. Landscape monitoring is generally based on continuous data sources and is often referred to as broad‐scale monitoring, macro‐systems ecology (Rose *et al*., 2015; Rose *et al*., 2017) or even just remote sensing (Pettorelli *et al*., 2014*a*; Pettorelli, Safi, & Turner, 2014b). This type of monitoring most commonly utilises the spatial technologies of remote sensing, geographical information systems (GIS), and environmental modelling (Turner, 2014). Landscape monitoring can also utilise aggregate outputs such as river flow monitoring to infer information over large areas. These methods often focus on reflectance measures that are observable from satellite imagery (Harwood *et al*., 2016) that act as surrogates (a variable with which the change that you are interested in strongly correlates) (Pettorelli *et al*., 2018) for a biological or environmental variable of interest (Marsett *et al*., 2006). It is important to note that there are many environmental variables that do not manifest as a change in spectral properties and so cannot be identified with remote‐sensing technologies, although other landscape monitoring techniques (modelling, GIS, aggregate information) may be appropriate in these situations.

A clear strength of landscape monitoring is its ability to collect information from remote and difficult‐to‐access areas. It commonly provides spatially continuous data (raster data sets) over large geographic areas at scales where ground‐based data collection is simply not plausible (Pettorelli *et al*., 2014*a*). Land cover (Scarth *et al*., 2015; Melville, Fisher, & Lucieer, 2019), ground cover (Bastin, 2014), vegetation mapping (Sparrow & Leitch, 2009), flooding, fire location, severity and frequency (Maier, Ludeker, & Gunther, 1999; Edwards, Russell‐Smith, & Maier, 2018), and vegetation structure (Gill *et al*., 2017; Scarth *et al*., 2019) are just a few environmental phenomena that are routinely correlated with changes in spectral reflectance. Calibration of surrogate measures and biological/environmental variables are often identified from previous targeted monitoring work (Bunce *et al*., 2007). However, there are many examples where field truth data are not used (Bunce *et al*., 2007) or remote sensing techniques are ‘truthed’ against other higher spatial resolution remote‐sensing techniques rather than actual biophysical properties (Hansen *et al*., 2013). Similar to surveillance monitoring, landscape monitoring is able to detect changes previously unexpected and/or unpredicted, and this ability is enhanced if integrated with other types of monitoring (Schmeller *et al*., 2017*a*).

Relationships developed between reflectance and variables of interest need to be validated using information that was not used to create these models along with accuracy assessments of resultant mapping (Congalton & Green, 2008). Examples such as the accuracy assessment of forest cover analysis (Bastin *et al*., 2017) and validation of crowdsourced cropland area analysis (Fritz *et al*., 2015; Lesiv *et al*., 2019) demonstrate this value. Most landscape monitoring uses information from either targeted monitoring or surveillance monitoring as validation data sets to inform, train, or test its procedures (Bastin *et al*., 2009; Huete, 2016; Pettorelli *et al*., 2016; Luque *et al*., 2018), although this can be hampered by poor field data quality. Landscape monitoring rarely distinguishes between the different forms of field monitoring programs that can be used as inputs for validation (Huete, 2016). Remote‐sensing techniques used in landscape monitoring can be applied consistently across vast areas, with some authors (Pettorelli *et al*., 2014*a*; Luque *et al*., 2018) claiming that remotely sensed data is the only way to obtain standardised biodiversity information over large areas in reasonable time periods. This spatial focus means that this type of monitoring is often conducted by national research infrastructures (Cleverly *et al*., 2019), or groups of international cooperation such as the Group on Earth Observation (earthobservations.org), NASA and other similar groups.

Advances in imaging availability and processing ability increasingly enable landscape monitoring to inform on temporal issues to a greater extent than previously possible (Pettorelli *et al*., 2018). The ability to examine large areas over regular time periods *via* satellite image archives allows for the detection of long‐term trends (Schmeller *et al*., 2017*b*) and is a clear strength of this form of monitoring (Kennedy *et al*., 2014; Estes *et al*., 2018). This is particularly valuable because it enables modellers to hindcast and check how models perform with historic data, providing greater confidence in their predictive power. No other form of monitoring enables the assessment of change in environments prior to the establishment of a monitoring program, making landscape monitoring an essential contributor to large‐scale monitoring programs. Drivers of change, however, cannot be identified using landscape monitoring alone. Change detected *via* landscape monitoring can be correlated with other environmental variables of interest obtained either by other forms of monitoring (Turner, 2014; Rocchini *et al*., 2018), or purposefully collected validation data. For example, Rocchini *et al*. (2018) regressed field heterogeneity (species diversity) against remotely sensed spectral heterogeneity to show that beta diversity increases as image spatial heterogeneity increases. With the move to larger‐scale continental and global analyses, requiring the integration of targeted, surveillance and landscape monitoring (Couvet *et al*., 2011; Ferreira, Rios‐Saldana, & Delibes‐Mateos, 2016) there will be an increased requirement for the scaling up of field information to validate imagery techniques with field‐based monitoring data.

## IV. WHY OUR SYSTEM NEEDS TO CONSIDER ALL MONITORING TYPES?

### (1) The need for integration across scales

As we have demonstrated, all monitoring types provide useful data for the scale and focus for which they are implemented, but none can address all questions required of a comprehensive monitoring system. This is because of the fundamental trade off between space, time and information content that exists across the targeted–surveillance–landscape spectrum. Recognition of this necessitates the integration of different types of monitoring.

Discussions advocating for the combination of remote sensing and field data are common. Since the 1990s, authors have highlighted the need closely to integrate good‐quality field data with remote‐sensing analysis (Roughgarden, Running, & Matson, 1991) to validate image‐analysis products (Marsett *et al*., 2006; Kao *et al*., 2012; Turner, 2014; Huete, 2016; Finer *et al*., 2018). Indeed, most authors conclude that both are essential to address large‐scale environmental questions (Dawson *et al*., 2011; Hampton *et al*., 2013; Gillan *et al*., 2014; Kennedy *et al*., 2014; Turner, 2014). Often field data is used as a ‘truth’ with which to conduct an accuracy assessment of image‐analysis products (Wallace *et al*., 2004; Congalton & Green, 2008; Hansen *et al*., 2013; Sexton *et al*., 2013). Site‐based information can also be ‘scaled up’ or extrapolated to areas between or beyond the specific sample locations to provide assessment over a much greater spatial area, to monitor environmental change continentally (Peters *et al*., 2014), or enable novel global analyses (Bastin *et al*., 2017, 2019). It is also common to compare and critique different types of field‐based monitoring for different applications (Vos *et al*., 2000; Wintle *et al*., 2010; Couvet *et al*., 2011). Lindenmayer *et al*. (2015) advocate for a balance between targeted and surveillance monitoring approaches, a view supported by Abbott & Le Maitre (2009) and by Wintle *et al*. (2010). Indeed, Wintle *et al*. (2010) consider the combination of multiple forms of monitoring of such great importance that they provide calculations to assist potential practitioners in determining how best to allocate resources between targeted and surveillance monitoring during the program design phase.

While many of these paired programs have been successful, we maintain that the most effective monitoring requires information that is integrated across all scales (Smith, 2002; Rose *et al*., 2017), involving information from targeted, surveillance and landscape monitoring. Ecological systems operate across many orders of magnitude along space and time dimensions and we therefore need information from all scales of monitoring (Belovsky *et al*., 2004; Lovett *et al*., 2007; Andersen *et al*., 2014), instead of the more traditional focus on information at a single scale. Therefore, aligned with the views of Eyre *et al*. (2011) and Scholes *et al*. (2008) we advocate for combining all three types of monitoring in a comprehensive monitoring system. A few programs have integrated information from each type of monitoring at smaller spatial scales, such as McCord *et al*. (2017) who utilise remote‐sensing data along with field‐based surveillance monitoring programs to inform rangeland management in the southern USA, but called for the inclusion of additional site‐specific targeted site information to provide the best combination of information. Vihervaara *et al*. (2015) also advocate for a system that utilises remotely sensed data, broad‐scale monitoring data from surveillance programs, and targeted studies from bird banding and citizen science bird observation programs to assist with ecosystem assessments. It should be noted that some monitoring programs are able to deliver on the aims of several types of monitoring. A good example of this would be the use of e‐bird (Sullivan *et al*., 2009) which was set up as a citizen science project and would most often be described as surveillance monitoring, but is regularly used in both targeted studies (Rodríguez‐Flores *et al*., 2019) and landscape studies (Muller, Veech, & Kostecke, 2018). Reinke & Jones (2006) suggest criteria that field‐based programs can include to enable integration with remotely sensed data. Although the combination of all three monitoring approaches has been discussed explicitly by some authors (Eyre *et al*., 2011; Turner, 2014), the benefits of providing information to answer all the types of questions posed to ecosystem monitoring has never been clearly addressed.

### (2) How information from these scales addresses key environmental change questions

The different types of ecosystem monitoring do have different strengths and some are better than others at addressing our key environmental change questions (see Table 2).

**Table 2:** Identifying the strengths of each monitoring type in relation to the types of questions that such a system should be able to address

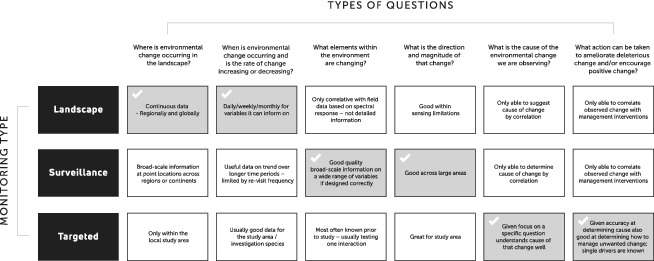

Landscape monitoring excels at addressing “*Where is environmental change occurring in the landscape?*” especially if the phenomena of interest correlate well with spectral reflectance signals sensed by satellite imagery. By providing continuous data across huge areas, we can determine where change is occurring at regional to global scales. Surveillance monitoring programs are also usually widespread across regional or national spatial scales and are able to provide useful information on representative areas, but they cannot be used to infer accurately the distribution of environmental change without extrapolation assisted by landscape monitoring. Targeted monitoring only provides information specific to a given site and, similar to surveillance, is not spatially continuous. Therefore, it cannot provide much information on the location of environmental change, although it is valuable as a robust source of calibration and validation data for landscape monitoring programs.

Landscape monitoring is also capable of addressing the questions “*When is environmental change occurring and is the rate of change increasing or decreasing?*”. The ability of common imagery types to provide information on a monthly, weekly or even daily basis facilitates investigations of ecological phenomena that are occurring over short time periods, including phenology events, events occurring as a result of seasonal variation, or the aftermath of extreme weather. The ability to hindcast also means that landscape monitoring provides one of the few mechanisms to investigate past environmental change (Clark *et al*., 2001). Targeted monitoring does collect information at a temporal scale such that short‐term variability can be disassociated from long‐term trends, but as discussed above, this type of monitoring has a limited spatial extent. The intensive nature of targeted field observations precludes any detailed temporal analysis over wide areas. Surveillance monitoring provides long‐term temporal trends over large areas, but the resources needed to monitor an area with sufficiently high temporal frequency are rarely available. Automation of some of the spatial elements of landscape monitoring is also leading to greater efficiencies in addressing some “when” and “where” questions.

By monitoring a broad suite of critical variables across regions, countries, and continents, surveillance monitoring programs are well placed to inform on “*What elements within the environment are changing?*”. Surveillance monitoring can accurately identify what is changing in the environment because it directly measures each variable, rather than relying on inferences based on correlations, as is the case with landscape programs. By tracking a wide variety of environmental variables, surveillance monitoring can also determine which components of the environment are subject to change, unlike landscape monitoring which is restricted to environmental phenomena that manifest as changes in spectral responses. Finally, because surveillance monitoring relies on field‐based methods, these programs are relatively sensitive to detecting change compared to landscape techniques. However, it is important to note that surveillance monitoring can be enhanced by combining data derived using this method with landscape monitoring to extrapolate where change is occurring. Targeted monitoring programs also provide good information on what environmental elements are changing, but with two major caveats: (*i*) change is usually only measured for a narrow range of variables; and (*ii*) the measured change will only be relevant to a restricted spatial area. Surveillance monitoring is also well‐equipped to determine “*What is the direction and magnitude of that change?*” across broad areas, including land types and jurisdictional boundaries. Both landscape and targeted monitoring programs can inform on magnitude and direction questions, however, as discussed above, targeted monitoring can only provide information for a restricted area, and landscape monitoring is limited to phenomena with a spectral response (or some surrogate thereof).

Given their hypothesis‐driven design, specific methods, and frequent sampling, targeted monitoring programs excel at determining “*What is the cause of the environmental change we are observing?*”. By focusing on a particular process‐based question, these programs can incorporate sufficient replication and power to inform on the causes of change. In addition, targeted programs can be used to compare areas with different land management or conservation strategies. As a result, they are the gold standard for informing on causation. Once a causal relationship is identified, the results of targeted monitoring can be integrated with other types of monitoring. For example, landscape‐monitoring data can be used to extrapolate process‐based mechanisms over similar areas, outside the original study site. Targeted monitoring is also uniquely positioned to inform on “*What action can be taken to ameliorate deleterious change and/or encourage positive change?”*. With careful program and experimental design, hypotheses about the impact of different management actions on known causes of change can be tested. Therefore, targeted programs are able to provide objective information on the most appropriate management technique from a range of options. Neither surveillance nor landscape‐based programs are able to determine the appropriateness of management actions in meeting diversity goals directly, and can only do so *via* a correlation between specific management interventions and measured responses.

### (3) Challenges to integration

It is worth clarifying that we are not arguing that all monitoring programs can or should be the same, rather, we argue that comprehensive monitoring programs rely on exploiting the strengths and complementary nature of multiple monitoring schemes. Individual programs might prefer one form more than another, but to monitor and manage regional to global areas we need to capitalise on the inherent strengths of each form of monitoring in a holistic system. Only then can monitoring programs help answer the six key environmental change questions we so often ask in ecological research. Whilst seemingly self‐evident, we reiterate that all types of monitoring benefit from clear objectives or direction setting (Wintle *et al*., 2010; Lindenmayer *et al*., 2014). Although objectives may differ between forms of monitoring, only when the objective of each monitoring program is clear are we able to understand how different types contribute to a holistic monitoring system.

A model where information from each of these scales is brought together in a cohesive monitoring network was first suggested by Scholes *et al*. (2008). Subsequently such systems are beginning to form with research infrastructure programs such as NEON (USA) (Kao *et al*., 2012; Peters *et al*., 2014), TERN (Australia) (Cleverly *et al*., 2019), and SAEON (South Africa) (van Jaarsveld *et al*., 2007), with international synthesis and cooperation being supported by GEO BON, the Global Environmental Research Infrastructure (GERI) group, the Forum on International Cooperation among Environmental Research Infrastructures (FIERI), and the Environmental Research Infrastructures (ENVRI) initiative.

Clearly, all three forms of monitoring are needed effectively to address all six questions we defined (see Table 2) over all possible temporal and spatial scales. Moreover, the complementary properties of these monitoring schemes mean that each can assist and support all other components of the system (see Table 2). Such a system, however, is only possible when managers and stakeholders take a collaborative approach to ecosystem monitoring. Specifically, this requires a strong appreciation for, and inclusion of diverse skill sets, and given the costly nature of ecosystem monitoring, an equitable division of limited resources. Teamwork, rather than competition, is crucial to success (Birnholtz, 2007). Collaboration enables the combination of information from each of these three types of monitoring. Conservation practitioners, policy officers, NGOs and researchers should all work together cooperatively to build this system (Pettorelli *et al*., 2014*a*). In order to develop a diverse, three‐tiered monitoring program, specialists in each type of monitoring need to come together and share their skills (Pettorelli *et al*., 2014*a*). It is simply not possible for any one person or group to develop the incredibly vast and divergent skill sets needed to create the complete and holistic monitoring network we have proposed here. For example, many landscape‐scale monitoring analyses are computationally intensive, requiring the use of dedicated software and computing hardware. In addition, the use of remotely sensed imagery and equipment typically requires specialised training (Roughgarden *et al*., 1991), although advances in technology are making these data increasing accessible to non‐specialists (Pettorelli *et al*., 2014*a*). This type of monitoring also is often reported in domain‐specific journals and conferences focused on the environmental applications of these technologies (Watson & Novelly, 2004; Pettorelli *et al*., 2014*a*) and is therefore under‐represented in traditional ecological reporting forums (Watson & Novelly, 2004). As a result, many traditional ecological studies are unable to integrate landscape‐level data into their own research or other monitoring programs. Surveillance and targeted monitoring require in‐depth knowledge on a range of field sampling methods and species identification and measurement techniques *in situ*, understanding of habitats and landforms and the types of processes (more so for targeted monitoring) that occur between an organism and its habitat. Similar to landscape monitoring, results of surveillance and targeted monitoring schemes are presented and published in venues not particularly accessible to spatial scientists. Ultimately, to realise a comprehensive three‐tiered monitoring program will require all conservation practitioners, policy officers, and researchers to combine their skills and provide clear avenues for effective communication whilst always valuing the contribution of their peers working under other monitoring paradigms.

All forms of ecosystem monitoring are relatively costly to implement and require sustained investment over long periods for maximum benefit (Lovett *et al*., 2007). When utilising the framework described herein it becomes apparent that argument as to what form of monitoring should be preferentially funded is moot. Far more important is the fact that all forms of ecosystem monitoring are critically underfunded (McDonald‐Madden *et al*., 2010), with many programs either halted or de‐funded before they have collected sufficient data to demonstrate their value. There is a fundamental disconnect between the rates of environmental change, which occur over decades and centuries (Andersen *et al*., 2014), and the common three to three‐to‐four‐year political and grant‐funding cycle. Additionally, ecosystem monitoring programs are politically easier to de‐fund compared to issues of health, education, and safety that the public identify with more immediately. Experts continue to suggest (and we very much agree) that governments should invest more in monitoring (Pereira *et al*., 2010), particularly considering the cost of inaction (Costanza *et al*., 1997).

## V. CONCLUSIONS

1. Maintaining ecosystem services is currently one of society's greatest challenges. To do that effectively we need ecosystem monitoring programs that can inform on different aspects of environmental change. Information from these monitoring programs is essential to successful land management actions. However, traditionally there has been little agreement about how to define different forms of ecosystem monitoring or how they can contribute to various management and research goals.
2. Herein we have provided clarity around a framework that can be used succinctly to articulate types of monitoring and their value in observing different forms of ecological change. We have identified three types of monitoring, namely landscape, surveillance and targeted monitoring, and explored each of their applications. We recommend this framework to assess current monitoring programs and to discuss and design effective future programs. This system is broad, clear, flexible, and can be used to investigate a wide range of changes in the environment at a variety of scales.
3. We have also detailed six key questions that ecosystem monitoring is expected to inform. Ecosystem monitoring questions relate to where and when change is occurring, what elements of the environment are changing, and the direction and magnitude of that change. Questions of why change is occurring, and how to ameliorate undesirable change are also critical to our understanding of environments. We have documented how each form of ecosystem monitoring contributes to our ability to inform on these questions. In this context, we identified the strengths and weaknesses of each type of monitoring.
4. We have established it is essential that all three types of monitoring be implemented together in order for all forms of ecological questions to be addressed. With well‐designed surveillance monitoring complementing both targeted and landscape‐wide programs, we can arm ourselves with the information necessary to address some of our greatest environmental challenges.
5. Finally, we advocate for renewed cooperation and collaboration between practitioners of each form of monitoring, and for them to work together to provide society with this crucial information. We encourage them to articulate their value, and the value of all types of ecosystem monitoring in a holistic rather than competing manner. Only when that happens will it be possible for society to realise the essential value of ecosystem monitoring programs.

## AUTHOR CONTRIBUTIONS

B.D.S. developed the idea, B.D.S. conducted the literature review, all authors contributed ideas, B.D.S. wrote the initial draft, and all authors provided assistance and substantial revisions.
